# Intersection and Complement Set (IACS) Method to Reduce Redundant Node in Mobile WSN Localization

**DOI:** 10.3390/s18072344

**Published:** 2018-07-19

**Authors:** Muhammad Zar Mohd. Zaid Harith, Noorzaily Mohamed Noor, Mohd. Yamani Idna Idris, Emran Mohd. Tamil

**Affiliations:** Department of Computer System and Technology, Faculty of Computer Science and Information Technology, University of Malaya, Kuala Lumpur 50603, Malaysia; mzar_um93@siswa.um.edu.my (M.Z.M.Z.H.); emran@um.edu.my (E.M.T.)

**Keywords:** computation cost, communication cost, energy, localization, Monte Carlo, WSN

## Abstract

The majority of the Wireless Sensor Network (WSN) localization methods utilize a large number of nodes to achieve high localization accuracy. However, there are many unnecessary data redundancies that contributes to high computation, communication, and energy cost between these nodes. Therefore, we propose the Intersection and Complement Set (IACS) method to reduce these redundant data by selecting the most significant neighbor nodes for the localization process. Through duplication cleaning and average filtering steps, the proposed IACS selects the normal nodes with unique intersection and complement sets in the first and second hop neighbors to localize the unknown node. If the intersection or complement sets of the normal nodes are duplicated, IACS only selects the node with the shortest distance to the blind node and nodes that have total elements larger than the average of the intersection or complement sets. The proposed IACS is tested in various simulation settings and compared with MSL* and LCC. The performance of all methods is investigated using the default settings and a different number of degree of irregularity, normal node density, maximum velocity of sensor node and number of samples. From the simulation, IACS successfully reduced 25% of computation cost, 25% of communication cost and 6% of energy consumption compared to MSL*, while 15% of computation cost, 13% of communication cost and 3% of energy consumption compared to LCC.

## 1. Introduction

A typical robot localization depends on the information from common sensors [1] such as laser range scanner and ultrasonic which has a high cost of remote deployment. Contrarily, Wireless Sensor Network (WSN) localization utilizes wireless communication features to estimate position information. WSN localization is a process to estimate location information of unknown or blind nodes in a wireless network. Therefore, it can be useful in many areas such as military surveillance, precision agriculture, health monitoring and environmental monitoring. The communication features of WSN localization include hop count, propagation time, received signal phase/angle and received signal strength. These features are then forwarded to estimation modules such as the Kalman Filter (KF) and Monte Carlo algorithm. The estimation algorithm is used to accurately estimate the position information based on the data from the features.

Apart from the wireless communication features and the estimation module, another unique characteristic of WSN localization is its capability to share information among communication devices. Consequently, nodes with known location can be used to estimate the location of blind nodes. This can be beneficial when information from the selected features are insufficient to estimate the position information. However, the estimation of blind node location from the nodes with known location normally involves a large number of nodes. Involving a large number of nodes can increase the estimation accuracy but at the expense of computation and communication costs. Communication and computation costs are the two main components that affect energy consumption in WSN devices [2]. The communication cost includes the energy consumed during the data transmission and reception, while the computation cost is the energy consumed to execute algorithms in the device or node. A high communication and computation cost will rapidly deplete the energy and shorten the node lifetime. For this reason, a trade-off between accuracy and energy consumption should be taken into consideration in development of WSN localization algorithm.

In this paper, a Monte Carlo-based localization algorithm will be studied. Monte Carlo-based localization, also known as Particle Filter (PF) localization is chosen due to its efficiency, lightweight, and adaptability in a dynamic topology environment like mobile WSN environment [3]. This study will be focusing on reducing the computation, communication, and energy costs of the localization process, while maintaining its accuracy. Computation and communication costs are important factors in determining the node lifetime, but limited studies have highlighted the respective issues. Based on our review on recent Monte Carlo-based localization, only Low Communication Cost (LCC) scheme [4] comprehensively addresses the issues. The LCC scheme utilizes adjacency matrix, set theory and intersection set to remove unnecessary redundant data. Their result shows that their method reduces the communication cost, which also reduces the energy consumption while maintaining the accuracy of the localization estimation.

Inspired by the LCC, we propose a scheme called Intersection and Complement Set (IACS). Both the LCC and the IACS are based on the Monte Carlo localization algorithm that includes both first hop and second hop data to estimate the blind node location. However, the LCC scheme only considers the intersection set, which only reduces the redundant data in the first hop nodes. Unlike the LCC scheme, the IACS scheme utilizes both intersection and complement sets. Since second hop data is used, the complement set in IACS is expected to find other possible redundant data that can be safely discarded. IACS also introduces two major stages named as duplicate cleaning and average filtering. In the duplicate cleaning stage, neighbor nodes that have the same intersection set or complement set are discarded from the localization estimation. Then, the average filtering stage will only select neighbor nodes with a higher number of elements in the intersection or complement sets. This will further reduce the excessive data, which is not needed to estimate the location information.

To ensure the reliability of our proposed method, we evaluate our proposed method with Mobile and Static sensor network Localization* (MSL*) and LCC scheme. In the evaluation, all methods are tested in various scenarios that consist of default parameter, different maximum number of samples, different nodes mobility speed, different degree of irregularity, and different node density. Their performances are evaluated and compared in terms of localization accuracy, closeness value, computation cost, communication cost, and energy consumption.

The rest of this paper is organized as follows: Section 2 reviews the literature on WSN Localization. Section 3 discusses the problem analysis on the benchmarks. Section 4 describes the details of the proposed IACS method and the experimental setup for the simulation. Section 5 presents the description of the evaluation metrics follows with the evaluation results. Discussion and conclusion follows in Section 6 and Section 7.

## 2. Literature Review

Conventionally, the blind node location can be estimated using GPS embedded on the devices [5]. This satellite-based positioning system is accurate, but its reliability is susceptible to the weather, presence of obstacles, position of satellites and the hardware’s limitation. Furthermore, the usage of GPS would increase the overall cost due to the requirement of additional hardware. Alternatively, WSN localization uses neighboring nodes, nodes that obtained their location from data shared by their respective neighbors. The usage of neighboring nodes would reduce the dependency on the GPS.

Two important attributes in WSN localization are wireless communication feature and estimation algorithm. Wireless communication feature is a feature, which contains an informative and non-redundant data extracted from an initial set of measured data using wireless devices. The estimation algorithm, on the other hand, is a method to estimate the position information based on the extracted features. The following section will discuss these two attributes and follow with Monte Carlo-based localization methods.

### 2.1. Wireless Communication Feature

In WSN localization, wireless communication features are used to provide input to the estimation algorithm. Wireless communication features have a distinctive characteristic of a wireless device that contains measurement that is essential for position estimation. Typically, the distance measurement is extracted from these communication features. The communication features include hop count, propagation time, received signal phase/angle and received signal strength. In the hop count, the distance is measured from the number of node-to-node hop count. Propagation time utilizes the Time of Arrival (ToA), Time Difference of Arrival (TDoA), and Return Time of Flight (RToF), while received signal phase/angle uses Angle of Arrival (AoA), RSP and interferometry techniques [6]. In received signal strength [7], RSS and Channel State Information (CSI) techniques are used. These features are then sent to a localization estimation algorithm to estimate the actual position of a blind node.

### 2.2. Localization Estimation Algorithms

Localization estimation algorithm is used to estimate the position information of a blind node based on partial observations of other measurements. Two of the most widely used estimation algorithms in localization are KF and PF/Monte Carlo Algorithms. Both KF and PF algorithms are based on Recursive Bayesian estimation that estimates an unknown probability density function recursively over time using incoming measurements and a mathematical process model. The KF achieves this goal by deriving analytics equations based on multivariate normal distributions and linear projections. For this reason, KF is excellent in estimating information in linear and normal distribution environments [8,9,10,11,12,13]. Conversely, PF utilizes a set of discrete points or particles. The particles represent the distribution of likely states, with each particle representing a possible state. Therefore, PF is more efficient in non-linear and non-Gaussian environments. Furthermore, compared with the traditional trilateration localization method, PF is more robust and has less localization error in each iteration [14]. Taking into consideration the non-linear model of a number of mobile WSN, this paper concentrates on the PF localization method also known as Monte Carlo localization (MCL) [15]. The following section will discuss the details of Monte Carlo-based localization methods.

### 2.3. Monte Carlo Based Localization Method

Monte Carlo-based localization algorithm normally starts with a uniform random distribution of particles over a configuration space. If the node moves, the particles shift to predict its new state. When new data is sensed, the particles are resample based on Recursive Bayesian estimation. The algorithm’s goal is to converge the particles toward the actual position of the node. From the convergence, location information can be extracted.

The preliminary algorithm of the Monte Carlo-based localization is MCL [15]. It estimates the blind node location based on the data from seed nodes. A seed node is a node that is equipped with positioning hardware such as Global Positioning System (GPS). However, the MCL is able to achieve a good estimation of the location information; the high dependency on the positioning hardware increases the costs of the localization process. Therefore, Mobile and Static sensor network Localization (MSL) and MSL* are proposed to reduce the dependency on the seed nodes. The MSL and MSL* improve the MCL by taking the location information not only from the seed nodes but also from neighboring normal nodes [16]. Normal nodes are the nodes that contains location information estimated from the seed nodes. The inclusion of normal nodes in this cooperative method reduces the seed nodes cost and improves the localization accuracy within the range of its radio signal. The accuracy is improved due to additional information received from the normal nodes instead of solely depending on the seed nodes.

Looking at the advantage of MCL, MSL and MSL*, other Monte Carlo-based localization methods are proposed by prior researchers. Among them are Monte-Carlo Box (MCB), VMSL, VMSL*, WMCL, and LCC. The focus of these methods is to improve the sampling efficiency while retaining the accuracy of the prior Monte Carlo methods. The sampling efficiency looks into sampling nodes with significant information instead of taking all information from the adjacent nodes. The purpose of selective sampling is to reduce execution time, computation cost, and communication cost of a localization process. Each of the proposed methods have their own unique properties to improve sampling efficiency. For example, MCB [17] uses square bounding box to optimize the number of valid samples from the seed nodes. Samples are selected from the intersection area of the bounding box between the current and the previous location of the seed nodes. On the other hand, VMSL, VMSL* and WMCL [18] introduce a technique that reduce the MCB bounding box size. The research of sampling efficiency continues with the introduction of the LCC [4] scheme. The LCC utilizes an adjacency matrix and set theory instead of a bounding box method as in prior MCB-based methods. From the result, it can be seen that LCC outperformed MCB. This is because LCC reduces additional data broadcast cost that is needed by the MCB-based method to create the bounding box. 

Considering the advantage of the LCC scheme over other sampling methods, LCC is selected as the benchmark in this paper. To make this paper self-contained, an extensive simulation on both MSL* and LCC is done to investigate the research gap. The investigation is explained in the next section.

## 3. Problem Analysis

The simulation details of MSL* and LCC are explained in this section. For this research, Netbeans (netbeans.org) is used for the simulation of MSL*, LCC and IACS. Netbeans is an Integrated Development Environment (IDE) for Java language. The base source codes for the simulation have been acquired from the original author.

**Figure 1:**
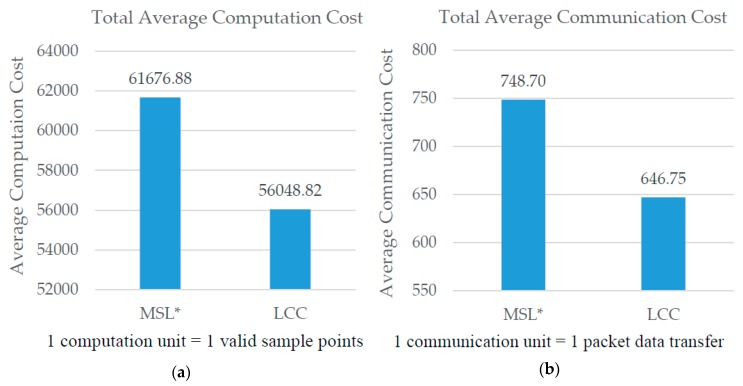
(**a**) Average computation cost of MSL* and LCC. (**b**) Average communication cost of MSL* and LCC.

Sensor nodes *N* are arbitrarily scattered throughout the 2-dimensional Euclidean space (2E) to create the WSN environment. The parameters of our experimental setup are shown as in Table 1. LCC [4] reduces the communication and computation cost of the localization process in MSL by reducing the number of nodes used during the localization process. Figure 1a,b shows the comparison of overall computation and communication costs between MSL* and LCC. Based on these results, reducing the number of neighbor nodes used for the localization process contributes to a significant reduction of computation and communication costs.

Further study on LCC is done to detect any research gaps. As a start, a simulation to detect redundancy on the LCC node selection method is performed. The simulation is based on the experimental setup in Table 1 including the list of all the intersection sets of each blind node neighbors. 

LCC [4] filters the neighbor for each node based on intersection sets. Table 2 shows the examples of the filtered neighbor nodes (Id), selected based on their respective list of neighbor sets and intersection sets. Based on the table, there are duplicated intersection between node 8 and node 9. This shows that node 8 and 9 share the same information for the filtering method in LCC. Therefore, a simulation to detect these duplicates is done using the experimental setup in Table 1.

**Figure 2:**
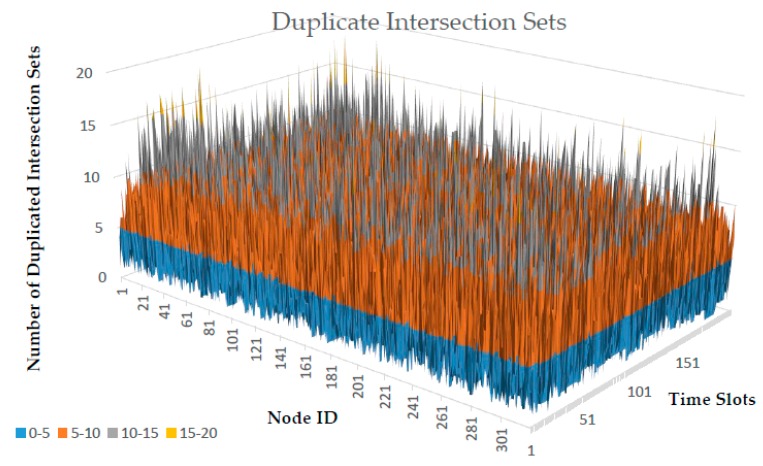
Duplicate Intersection Sets. The surface chart represents the example of duplicate intersection detected by each node for each time slot obtained from the simulation. The legend shows the range of duplicated intersection sets detected, defined by different colors. The results vary depending on the location and movements of each node during the simulation.

Table 3 shows the tabulated version of duplicate intersection sets in Figure 2. Based on the simulation, the basic statistics of duplicated intersection sets detected for 320 nodes within 200 time slots are summarized in Table 4. The maximum duplication can reach up to 20, while zero (no duplicate) is the minimum. Each node has approximately an average of 5 duplicate intersection sets while the total duplication in each node that frequently occurred is 3.

In term of information sharing, even though the sampling process requires the data from both first and second hop neighbors, LCC only considers the total intersected neighbors between the blind node and its neighbors. Therefore, by also considering the total non-intersected neighbors shared between the blind nodes and their neighbors, more nodes for the localization process can be filtered. For these reasons, IACS is proposed to safely discard these redundant data in order to minimize the localization cost. IACS considers both intersection and non-intersection sets to further reduce the redundancy (Section 4). The complement set is included in the proposed method to remove the redundant data from the neighboring node that is outside of the blind node signal range.

## 4. Proposed Method

The following sections explain the implementation of the IACS method in the MSL* localization algorithm. The simulation consists of three stages: initialization, IACS, and location estimation.

### 4.1. Initialization

**Figure 3:**
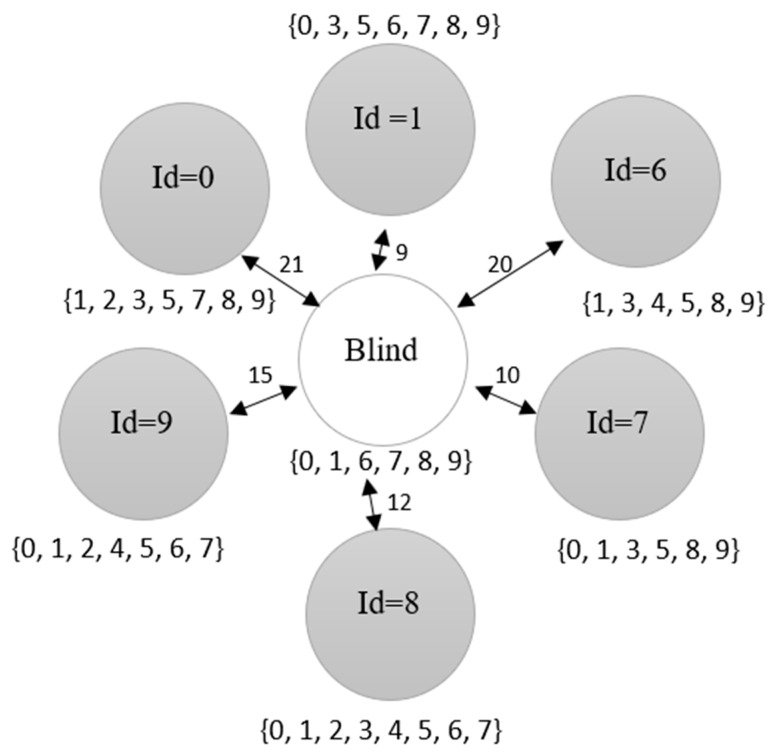
Example scenario of a blind node surrounded by a set of neighboring nodes. Each node has its own neighbor set. The arrows show the distance in meters (*m*) between the neighboring nodes and the blind node.

During the initialization, sensor nodes are scattered randomly within a defined area without any information about their location. Then, the relation between the nodes in the network are established according to their distances from each other. The distances are used to group the nodes as a first hop neighbor, second hop neighbor or out of range. For the simulation, the distance is measured in meters (*m*). Figure 3 shows a blind node surrounded by a set of neighboring nodes labelled with an identity number (Id). At this stage, only seed nodes will broadcast their location since the location of the other nodes is still unknown. The closeness value and the samples for each node are also initialized and explained in the location estimation section (Section 4.3).

### 4.2. IACS

**Figure 4:**
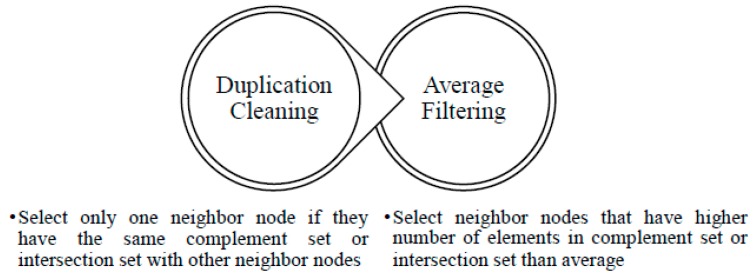
Two main steps in IACS method to remove the redundant neighboring node.

There are two main steps in IACS, namely duplication cleaning and average filtering, as depicted in Figure 4.

#### 4.2.1. Duplication Cleaning

In this step, we compare the intersection and complement sets of each neighbor node with each other to find any duplication set. If the duplication set exists between the neighboring nodes, only the node with the shortest distance to the blind node is selected to avoid delay when connecting to far-away nodes. Therefore, less communication between the nodes is needed for the localization process when the duplicated data are ignored.

For example, Figure 3 shows the neighbor set of the blind node is {0, 1, 6, 7, 8, 9} and the neighbor set for node Id = 0 is {1, 2, 3, 5, 7, 8, 9}. The intersection set is the elements of the neighbor node’s neighbor set that intersects with the blind node’s neighbor set. Therefore, the intersection set of blind node and node Id = 0 is selected as {1, 7, 8, 9}. The complement set on the other hand is the elements of the neighbor node’s neighbor set that do not intersect with the blind node’s neighbor set, which result in the selection of {2, 3, 5}. Based on Figure 3, Table 5 is tabulated. Table 5 shows that the complement set of nodes 1 and 7 are duplicated. For this case, node 7 is removed as the distance of node 7 to the blind node is further than the distance of node 1 to the blind node. Later, node 9 is removed due to duplication of the intersection set with node 8 and it has a further distance to the blind node than node 8.

#### 4.2.2. Average Filtering

Since the sample’s weight is based on the first and second hop neighbors from the sample’s points (including neighbors to the blind node neighbor), we consider the complement elements of the neighbor set between the neighbor node with the blind node to remove nodes that provide data less than the second hop neighbor. Total elements from both intersection and complement sets are calculated for their average. If the average contains decimals, the largest (closest to positive infinity) integer value that is less than or equal to the average is selected. If the total elements in the intersection set or complement set is lower than the average, they are considered as out of range, thus they are removed from the localization process. From the scenario given in Figure 3, neighboring node 6 is removed because the total elements in the intersection set are lower than the average of the intersection elements of 4. Then, node 1 is removed (Table 6) since the total elements in the complement set are lower than the average elements of three.

This step can prevent the blind node from communicating with nodes that provide redundant and less significant information for the localization process. Therefore, the cost of computation and communication are expected to decrease in IACS because fewer nodes are used without ignoring the important data for the localization process.

### 4.3. Location Estimation

The estimation process of the nodes location is computed according to [16]:

(1) $$e\left(x\right)=\;\frac{\sum_{i=1}^{N}s_{i}(x)\;w_{i}\left(p\right)}{\sum_{i=1}^{N}w_{i}\left(p\right)}$$

(2) $$e\left(y\right)=\;\frac{\sum_{i=1}^{N}s_{i}(y)\;w_{i}\left(p\right)}{\sum_{i=1}^{N}w_{i}\left(p\right)}$$

where, *e*(*x*) and *e*(*y*) represent the estimated coordinates for node *p*(*x,y*). *S* denotes the sample points of the current time, N is the current total number of the samples for node *p* and *w* represents the weight for sample *s* at the current time. When there is no new sample produced, the current estimation point is the same as the previous estimation point.

The resampling process is performed by the normal nodes to remove samples with lower weight due to a fixed sample number and duplication of these samples in the new sample set. This process includes inserting the current samples set into the new sample set containing the probability that corresponds to its weight. The sample with a smaller weight will have a lower chance to be selected and vice versa. The location estimation of a blind node is highly affected by the closeness value, sample number and weight of its neighbor nodes as explained in the coming subsection.

#### 4.3.1. Closeness

The quality of location estimation in MSL* can be measured using closeness [16] equation as follows:

(3) $$closeness_{p}=\;\frac{\sum_{i=1}^{n}w_{i}\sqrt{{\left(x_{i}-x\right)}^{2}+{\left(y_{i}-y\right)}^{2}}}{N}$$

where *N* is the number of samples for node *p*, $\left(x_{i},y_{i}\right)$ represents the coordinate of the *i*-th sample (*i* = 1,2,…,*n*), $w_{i}$ represents the weight of the *i*-th samples, and (*x*,*y*) is the estimated location for node *p*. Closeness is measured when samples for the node in the current time are available, else, the closeness value is set to ∞. The closeness value of seed nodes is fixed to 0 while for others the value should be greater than 0. This indicates that the accuracy of the localization is high when the closeness value is small. The closeness value and location data of each node will be updated during the localization process once the location data and the closeness value of the seed nodes are broadcasted to their neighbors.

#### 4.3.2. Sampling

For location estimation, each node generates new samples from the current sample. The node will choose a random point within the radius $v_{max}+\;\alpha$ from the current sample [16] as shown in the given equation:

(4) $$p\left(S_{t}|S_{t-1}\right)=\;\{\begin{array}{c} \frac{1}{\pi {\left(v_{max}+\;\alpha \right)}^{2}}\;\;\;\;\;if\;d\left(S_{t},S_{t-1}\right)\leq \;v_{max}\\ \;\;0\;\;\;\;\;\;\;\;\;\;\;\;\;\;\;\;\;\;\;\;if\;d\left(S_{t},S_{t-1}\right)>\;v_{max}\;\; \end{array}$$

$d\left(S_{t},S_{t-1}\right)$ represents the distance between the locations of a sample at time *t* and *t* – 1 while $v_{max}$ is the maximum speed of a node. α is the variability for choosing new samples and α must not be too low so that it can provide variability in sampling even when the node’s speed is slow. In this paper, we set α=0.1r that is determined empirically as in MSL* [16], where *r* is the radio range radius of the sensor nodes. The sample also must be within the $v_{max}$ radius for mobile nodes.

The weight of each sample is determined by the product of partial weights, $w_{s}^{'}\left(q\right)$ [16] as described in the following equation:

(5) $$w_{s}\left(p\right)=\;\prod_{q=1}^{k}w_{s}^{'}\left(q\right)$$

*k* represents the number of neighbors in the first hop and second hop. *q* is a neighbor of node *p*. 

Partial weights, $w_{s}^{'}\left(q\right)$ that corresponds to neighboring seed nodes are set to 1 if d(s,q)≤r for first hop seed nodes and r≤d(s,q)≤ 2r for second hop seed nodes. For neighboring normal nodes, *q* in the first and second hop, the partial weights $w_{s}^{'}\left(q\right)$ can be calculated according to (6) and (7) respectively as follows:

(6) $$w_{s}^{'}\left(q\right)=\;\underset{q_{i}}{\sum }w\left(q_{i}\right),\;\;where\;d\left(s,q_{i}\right)\leq r+\;v_{max}$$

(7) $$w_{s}^{'}\left(q\right)=\;\underset{q_{i}}{\sum }w\left(q_{i}\right),\;where\;\;r-v_{max}\leq d\left(s,q_{i}\right)\leq 2r+\;v_{max}$$

Sample *S* is kept if $w_{s}\left(p\right)$ is larger than the threshold value β in which, β depends on the total number of neighbors in first hop and second hop (*t*) of node *p*. The value of $\beta ={\left(0.1\right)}^{t}$ represents that the uncertainty will decrease when the number of neighbors increase, which is unique for each node. After the number of samples obtained is sufficient or at maximum, the weights are normalized to ensure their total is equal to one. The weight normalization for the *i*-th sample is given by:

(8) $$\frac{w_{i}\left(p\right)}{\sum_{j=1}^{N}w_{j}\left(p\right)}$$

where *N* denotes the total number of the sample used for node *p*.

### 4.4. Simulation Experimental Setup

The performance of MSL*, LCC and IACS are compared and evaluated with identical experimental settings in Section 3 (Table 1) and true node locations. Since the environment occurs on a flat surface, the coordinate is in (*x*, *y*). RSSI is applied according to Reference [19] to derive the Euclidean distance *d*, between the pair of nodes that overlap.

Each node is assumed to have a perfect circle radio range, *r*, for transmission and their distance with other nodes are known. All three localization methods are assumed to run simultaneously using the same true location. The node density (Nd) is the average density of all sensor nodes in the tested environment, while the seed density (Sd) is the average density of the seed nodes. The simulation is executed for 50 iterations (200-time slot in each iteration), where the estimated node locations and their true location are reset after each iteration to compare the average accuracy over time between the methods. In each time slot, each node speed varies from 0 to *maxv* and moves in different directions. The nodes are assumed to move according to the modified random waypoint model [20] where the pause time is set to 0 [21]. The mobility reduces the dependency on neighbor nodes with known location. Despite the number of seed numbers deployed in the network, the number of the localized node increases in each time slot as long there is a seed node in the whole network. Each node changes places in each time slot to open the opportunity to gain new samples from their new neighbors. Therefore, nodes in the network localize faster if more seed nodes are initialized.

## 5. Evaluation

The performance of all methods simulated in the experiment are measured in terms of total average of localization error, closeness value, communication cost, computation cost, and energy consumption. Both communication and computation costs depend on the nodes mobility model, where in our case, modified random waypoint has been used to simulate the environment. The modified random waypoint model has been extensively used in WSN and has been proven experimentally by many researchers such as in References [4,15,16,17,18].

Other than that, we investigate the maximum number of samples, maximum velocity of each nodes, degree of irregularity, and node density in the performances of IACS, MSL* and LCC. However, it should be noted that the performances of all methods are measured only at the normal nodes without including the seed nodes, which is similar to prior studies conducted in MSL* and LCC.

### 5.1. Localization Error and Closeness

The accuracy of the estimated location in the localization process can be described with localization error. The localization error per time is evaluated similar to the error calculation in MSL* wherein a low localization error represents high accuracy and vice versa. The estimation error can be measured [4] as follows:

(9) $$Error=\;\;\frac{1}{n}\overset{n}{\underset{i=1}{\sum }}\left|\left|e_{i}-l_{i}\right|\right|$$

where *n* is the total number of sensor nodes, $e_{i}$ represents the estimation while $l_{i}$ is the true location of the *i*-th node. 

For the closeness evaluation, the total average closeness value is obtained for every case tested according to Reference (3). A low closeness value represents a high quality location estimation and vice versa. The metric unit of estimation error used in this study is meter (*m*).

### 5.2. Computation Cost

The computation cost is the cost to process valid sample production, as has been elaborated in Section 4.3.2. The overall computation cost of the localization process increases for every valid sample taken from each blind node’s neighbors and is used for the weightage calculation of the blind node samples. Measurement of computation cost is stated in the following equation:

(10) $$comp\;unit=\overset{n}{\underset{i}{\sum }}s_{q_{i}}$$

*n* represents the total number of the first and second hop neighbors of the blind node, while *s* is the number of samples of each neighbor node *q*. One computation unit represents one valid sample point obtained from a neighbor node for the blind node sample’s weightage calculation.

Therefore, the computation cost depends on the number of neighbor nodes and the number of samples used for the localization process. MSL* requires a high computation cost due to the usage of all nodes in the network but is outperformed by MCL in terms of convergence speed and sampling [4]. LCC then manages to decrease the computation cost by reducing the number of nodes being used for localization. Thus, implementing IACS will further decrease the computation cost.

### 5.3. Communication Cost

Communication cost is the cost it takes to exchange messages between the blind node and neighbor nodes within the blind node range. The number of message exchanges between the blind nodes and its neighbors to obtain localization information can be used to evaluate the communication overhead [15,22]. The number of message or communication cost is calculated according to n × s + m, wherein n is the number of nodes in the network, m is the number of seeds and s is the number of samples maintained by each node [16]. The number of message is influenced by the number of seed nodes and normal node (multiplied with its sample number i.e., 50) in first hop and second hop neighbors of the blind nodes. For the energy consumption calculation, the number of message exchanges of each node is represented into bit as explained in Section 5.4. The number of message exchanges (i.e., the communication cost) affected the overall energy consumption.

### 5.4. Energy Consumption

Dissipation of energy in the radio transmitter includes energy to run radio electronics, a power amplifier, and in a receiver. A threshold, $d_{crossover}$ is set to determine whether Friis free space, $\varepsilon_{fs}$ represents ($d^{2}$ power loss) or multipath fading $\varepsilon_{mp}$ model ($d^{4}$ power loss) for channel model as in Reference [23]. Therefore, the energy used to transmit the *l*-bit message at distance d is:

(11) $$E_{Tx}\left(l,d\right)=E_{Tx-elec}\left(l\right)+\;E_{Tx-amp}\left(l,d\right)$$

(12) $$E_{Tx}\left(l,d\right)=lE_{elec}+l\varepsilon_{fs}d^{2},\;d<\;d_{crossover}$$

(13) $$E_{Tx}\left(l,d\right)=lE_{elec}+l\varepsilon_{mp}d^{4},\;d\geq \;d_{crossover}$$

(14) $$d=\;d_{crossover},\;d_{crossover}=\;\sqrt{\frac{\varepsilon_{fs}}{\varepsilon_{mp}}}=87.7m$$

To receive the message, the energy value is:

(15) $$E_{Rx}\left(l\right)=E_{Rx-elec}\left(l\right)=\;lE_{elec}$$

Electronics energy, $E_{elec}$, is a combination of several factors such as sensing and actuation activity, signal coding, modulation, filtering, transmission and reception of the signal. Amplifier energy, $\varepsilon_{fs}$ or $\varepsilon_{mp}$, depends on distance to the receiver and acceptable bit-error rate. The value for $\varepsilon_{fs}$, $\varepsilon_{mp}$ and $E_{elec}$ are fixed as in References [23,24]. These formulas have been used in References [25,26,27,28,29] to calculate WSN nodes energy in a cluster formation environment.

For the size of the l-bit message in (12), (13) and (15), the calculation of message size as in Pongle [22] is implemented. Messages exchange between the blind nodes with other nodes only occurrs when they are related. To exchange the messages, each node must have its own neighbor’s information and send the information to the other nodes that are related to them. The total number of message exchanges can be represented as the overall communication cost (Section 5.3).

**Figure 5:**
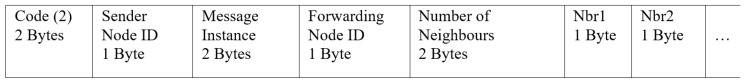
Neighbor Information Packet Structure.

One communication unit (message exchange) is represented by one packet data. This packet data is transferred or received by each node. Therefore, each number of messages exchanged between nodes in Section 5.3 is represented in bit for the energy consumption calculation. The structure of the packet data for the neighbor information that was used to calculate the size of the message sent and received is shown in Figure 5. However, the equation used bit unit for the packet size, therefore, 1 Byte = 8 bits.

In the simulation, the energy consumption of data transfer is calculated only from the blind node. As previously explained, the total energy consumption of each node is $E_{Tx}+E_{Rx}$ ((12), (13) and (15)) which depends on the total size of messages transmitted and received by each node per time slot.

### 5.5. Simulation Results

MSL*, LCC, and IACS are simulated in the same environment settings to enable a fair comparison between them in Section 5.5.1. From the simulations, the effects of the maximum number of samples, maximum velocity of mobile sensor node, degree of irregularity, and node densities are also studied and analyzed in the following sections. The graphical representations are based on the default parameters stated in Section 3 (Table 1). The default parameter was marked with ‘×’ on the x-axis in the figures for each result.

#### 5.5.1. Default Parameter

**Figure 6:**
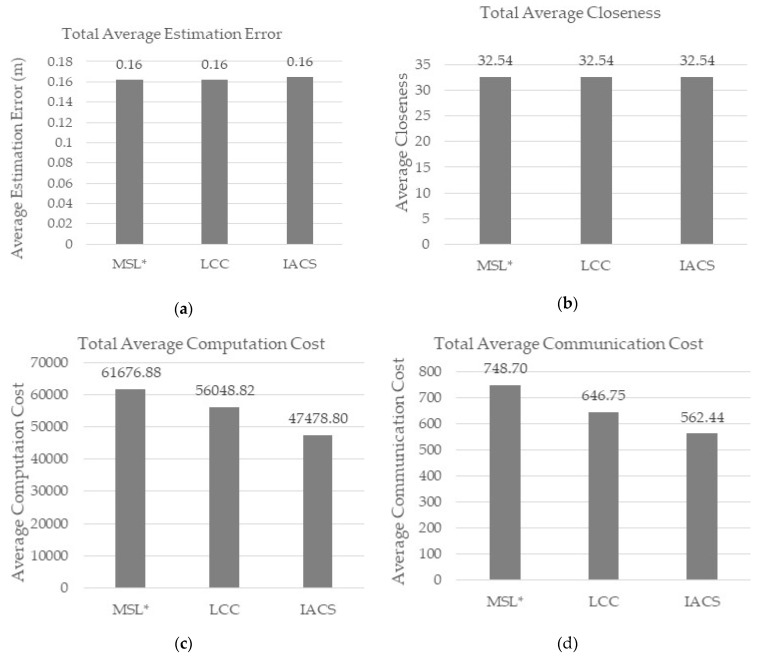

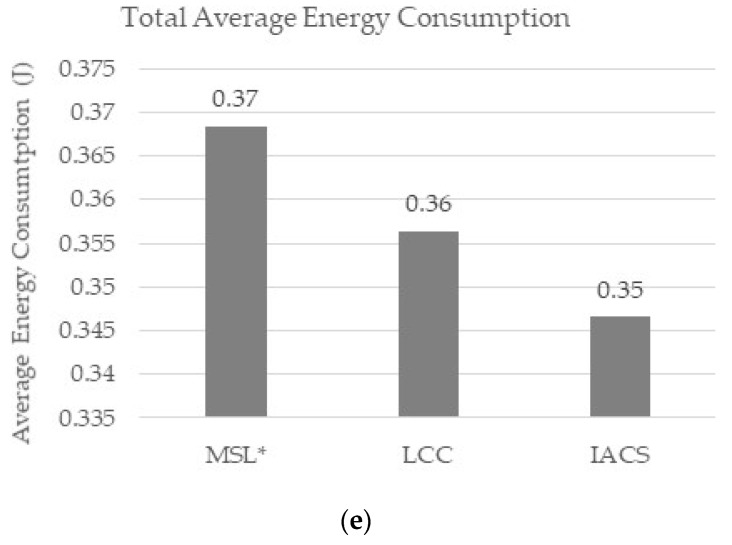
(**a**) Total Average Estimation Error, (**b**) Total Average Closeness, (**c**) Total Average Computation Cost, (**d**) Total Average Communication Cost, and (**e**) Total Average Energy Consumption for Default Parameter Scenario.

Using the default parameters, IACS successfully improved the computation, communication, and energy (Figure 6c–e) costs required for 50 iterations while maintaining the estimation error and closeness (Figure 6a,b) value for each normal node as in MSL* and LCC. Based on the figures above, IACS reduced 25% of computation cost, 25% of communication cost and 6% of energy consumption compared to MSL*, while 15% of computation cost, 13% of communication cost and 3% of energy consumption compared to LCC. The percentage is the difference between the value of each IACS evaluation metrics with respect to MSL* and LCC. For example, the difference percentage between IACS (562.44) and LCC (646.75) communication cost in Figure 6d: ((646.75 – 562.44) ÷ 646.75) × 100 = 13.04 ≈ 13%.

#### 5.5.2. Effect of Maximum Number of Samples

**Figure 7:**
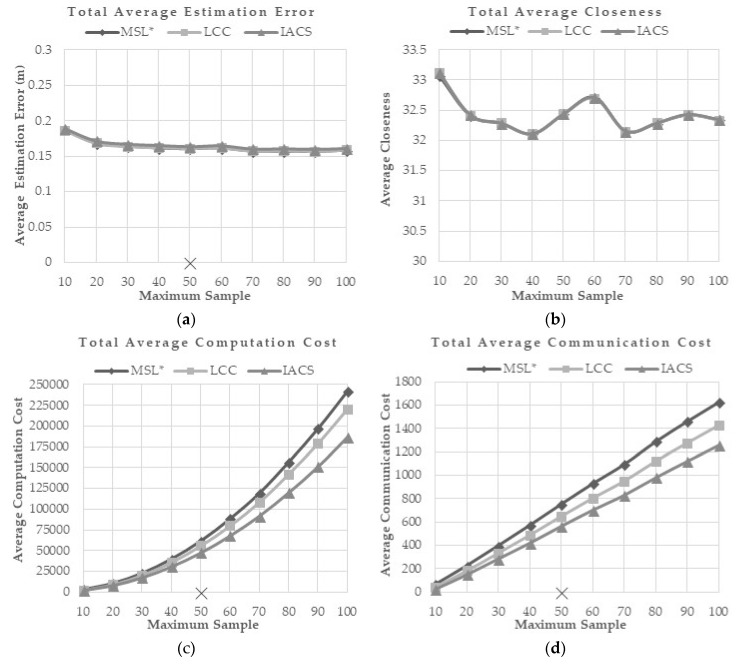

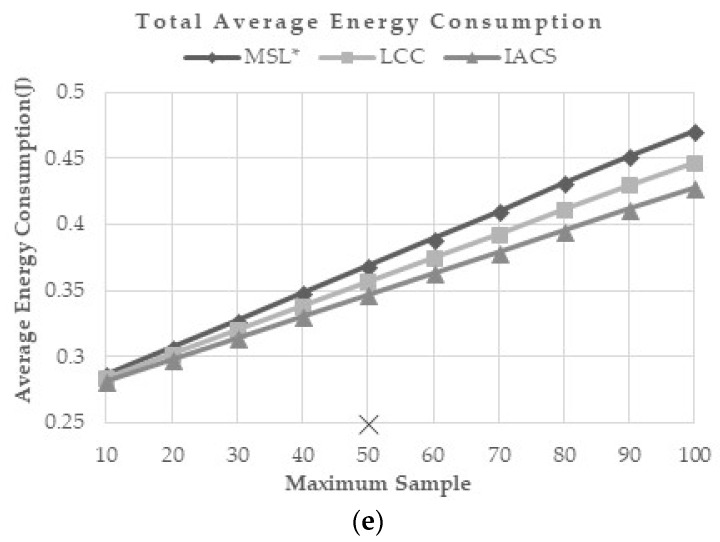
(**a**) Total Average Estimation Error, (**b**) Total Average Closeness, (**c**) Total Average Computation Cost, (**d**) Total Average Communication Cost, and (**e**) Total Average Energy Consumption for Different Max Samples Scenario.

This evaluation is done to study how the proposed method reacts to a different maximum number of samples. The maximum sample number used in previous researches is 50. In this evaluation, maximum number of samples varied between 10 to 100 and the effects of these variations on MSL*, LCC and IACS are presented in Figure 7a–e. Generally, a higher maximum sample number has little effect on the localization accuracy and overall closeness value. However, it increases the computation, communication and energy consumption.

From the simulation, although the communication, computation, and energy costs increase, IACS consistently shows the least costs compared to MSL* and LCC. However, similar accuracy and closeness value are observed between the methods without obvious changes when the maximum number of samples is increased.

#### 5.5.3. Effect of Mobility Speed

**Figure 8:**
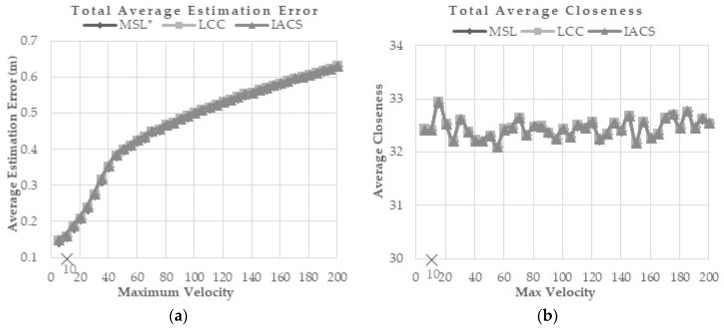

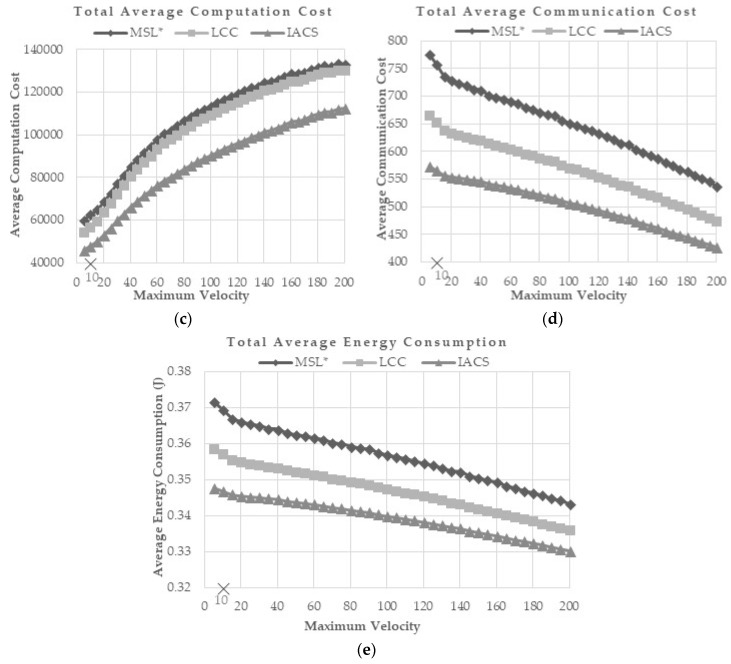
(**a**) Total Average Estimation Error, (**b**) Total Average Closeness, (**c**) Total Average Computation Cost, (**d**) Total Average Communication Cost, and (**e**) Total Average Energy Consumption for Different Maximum Velocity Scenario.

This evaluation is performed to observe how well IACS reacted under different maximum velocity compared with MSL* and LCC. To simulate a highly dynamic topology network, the modified random waypoint mobility model [21] and zero pause time are implemented into each of the nodes. The nodes in the network are assumed to move during each time slot at velocity, *v* = 1/*maxv*, where *maxv* is the maximum velocity of the sensor nodes. *maxv* values tested are from 5–200 while the default speed used in MSL* and LCC is 10 (×marked). According to the results, sensor node velocity is directly proportional to estimation error and computation cost, while it is inversely proportional to communication cost and overall energy consumption. As the velocity of the sensor node increases, the network topology changing rate will be faster. Therefore, each node will lose connection with its neighbor in a short time. Figure 8a,b shows that the error estimation and closeness value are not much different for the three methods.

From the simulation results shown in Figure 8c–e, IACS outperformed MSL* and LCC in terms of communication, computation, and energy costs while maintaining the similar error estimation and closeness value of normal nodes.

#### 5.5.4 Effect of the Degree of Irregularity

The degree of Irregularity (DOI) is the measurement of variation for range and direction of radio transmission. DOI is measured because the perfect circle of radio transmission that is assumed in the simulation does not represent the real value of radio transmission. The irregularity of radio range follows a normal distribution with mean *r* and standard deviation *σ* to allow greater variation [16]. Each sensor is assumed to have the same ideal radio range, *r*. *σ* is used to determine whether the sender or receiver is within *r*. The *σ* values used in the simulation are 0, 0.1, 0.2, 0.3, 0.4 and 0.5. Default DOI value tested on previous research is 0. A high value of DOI represents a high variation of the radio range transmission that may be caused by a high density of obstacle or climate changes in the real environment.

**Figure 9:**
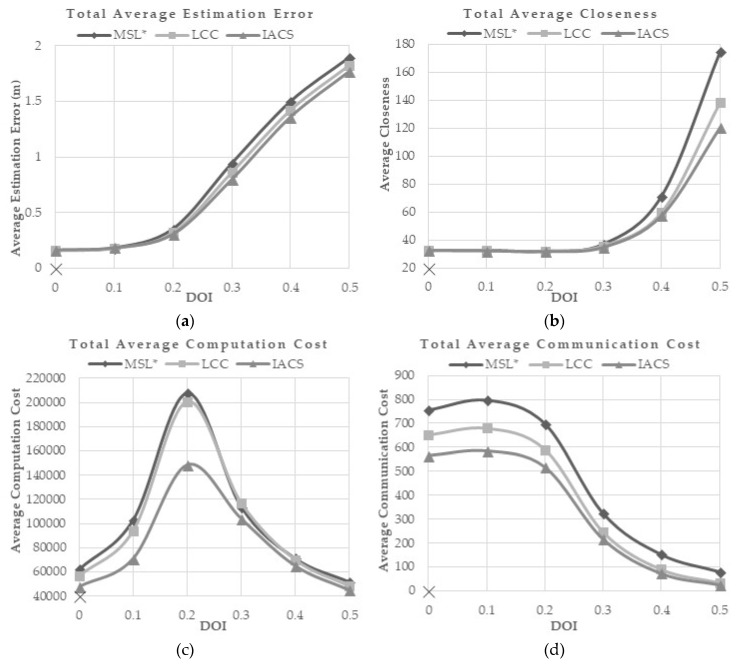

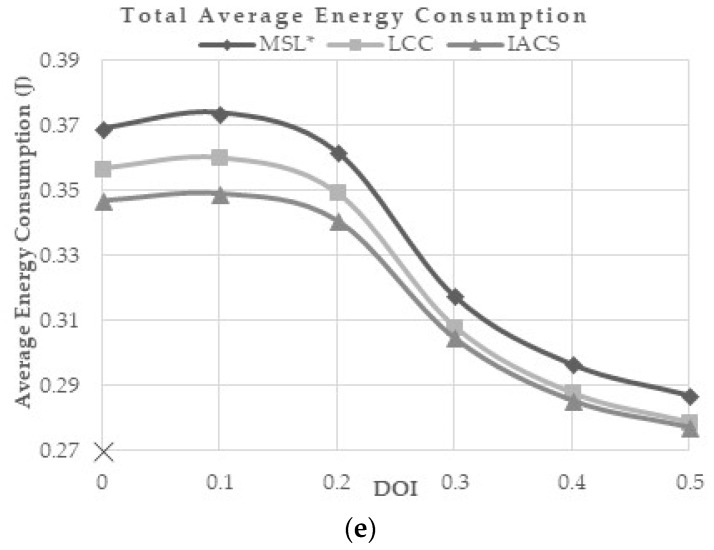
(**a**) Total Average Estimation Error, (**b**) Total Average Closeness, (**c**) Total Average Computation Cost, (**d**) Total Average Communication Cost, and (**e**) Total Average Energy Consumption for Different DOI.

Figure 9a,b shows that the average estimation error and the total closeness value of all methods are rapidly increased when the DOI value is above 0.2 and 0.3, respectively. However, the proposed IACS demonstrates the lowest average estimation error and the average closeness for every DOI compared to MSL* and LCC. This shows that IACS is more robust in the environment with a wider variation of radio signal transmission.

Figure 9c tells the average computation cost is at the maximum when the value *σ* = 2, where each blind node has the most number of neighbors. The computation cost is at the highest when *σ* ≥ 0.3, where the localization error starts increasing. When *σ* ≥ 0.4, the DOI is too high that some nodes may not relate to any nodes. Thus, the nodes will not have sufficient valid samples for the localization and results in high estimation error as shown in Figure 9a. The IACS method requires the least computation cost compared with the other methods.

As the number of neighbors in the range of blind nodes decreased, the communication cost and the energy consumption are decreased (Figure 9d,e). The proposed method requires the least communication and energy cost in different DOI.

#### 5.5.5. Effect of Node Density

**Figure 10:**
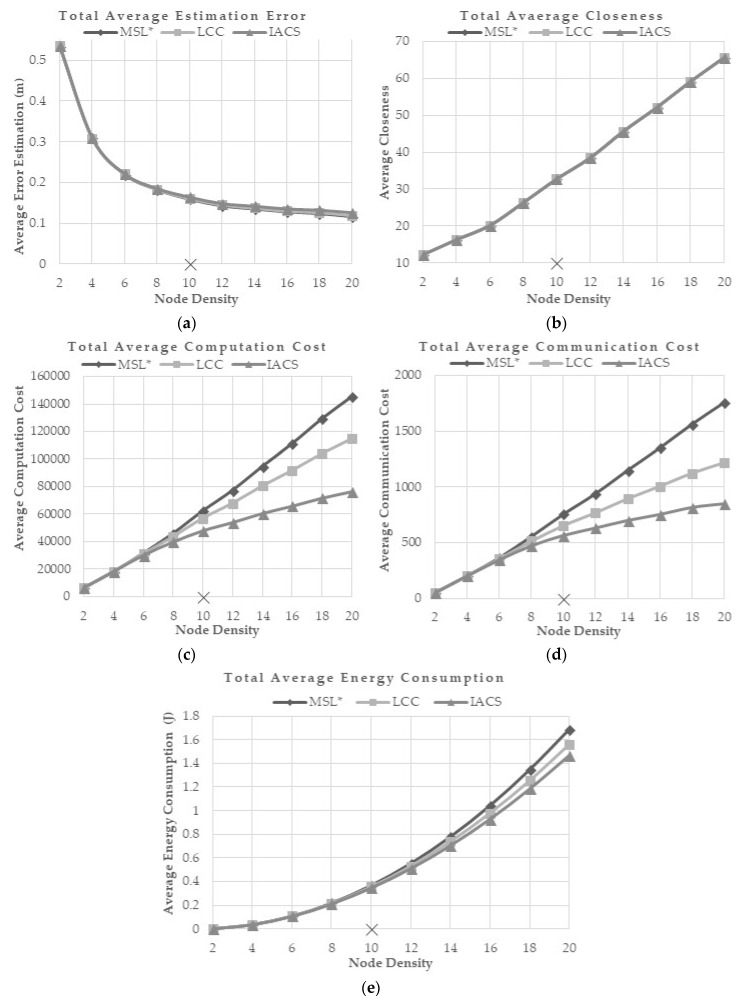
(**a**) Total Average Estimation Error, (**b**) Total Average Closeness, (**c**) Total Average Computation Cost, (**d**) Total Average Communication Cost, and (**e**) Total Average Energy Consumption for Different Node Density.

Node density represents the total density of nodes including both seed and normal nodes in the 500 × 500-unit area. A high node density represents a high number of sensor nodes in the environment that include both normal and seed nodes. The default node density parameter used is 10. This evaluation is performed to evaluate how well IACS with node density less and greater than 10. A density of 1 is equal to a total of 32 nodes. The seed nodes density tested for this evaluation is 1 in an environment of total nodes density ranging from 2 to 20. As the total number of nodes increases in the network, there will more nodes involved in the localization process that contribute to a better localization accuracy. However, this will increase the costs of the localization process. In that prospect, IACS provides the least costs of computation, communication and energy consumption compared to MSL* and LCC as the node density increases. IACS demonstrates a similar performance with MSL* and LCC in terms of average error estimation and closeness value wherein the average error estimation and closeness value increases with the increment of normal density (Figure 10a,b). For computation, communication and energy costs, the proposed IACS outperformed others by utilizing the least cost for different node density (Figure 10c,d).

The costs increase as the nodes density increases because more nodes are involved in the localization process as the number of normal nodes increase (Figure 10c–e).

Based on the simulation results, the proposed IACS reduced the amount of communication, computation and energy costs while maintaining its localization accuracy even at static mobility and mobile mobility with different DOI, normal density, seed density, maximum sample number and sensor nodes maximum velocity.

## 6. Discussion

In the localization process, IACS utilizes normal and seed nodes similar to LCC and MSL*. The proposed IACS improves LCC and MSL* by reducing the number of normal nodes used in the localization process. To reduce the number of normal nodes, IACS selects nodes with the total intersection set and complement set that is above the average value and one of the nodes that has a duplicate intersection set and complement set. This decreases the cost of computation, communication and energy consumption while maintaining the estimation accuracy as in LCC and MSL*.

Based on the simulation results, the number of valid samples used for localization can affect the costs of localization and the accuracy slightly. For example, utilizing a low number of samples will slightly decrease the accuracy and costs of localization while utilizing a high number of samples will increase the accuracy and cost of localization. However, the overall cost of computation, communication, and energy consumption is inversely proportional to the total number of samples. The default maximum number of samples in MCL, MSL* and LCC was set to 50 since it is proven experimentally as the optimum number of samples needed for localization in MCB [17]. The simulation results show that no obvious improvement is observed for all methods in terms of localization accuracy when the maximum number of samples is equal and larger than 50. For the cost of computation, communication, and energy consumption, IACS shows the least values compared with LCC and MSL* while maintaining the localization accuracy even with high sample numbers produced for the localization process.

Localization accuracy can be improved in a mobile WSN environment. Blind nodes can obtain more location information from more nodes for the localization process. However, accuracy can only improve when the sensor node velocity is slow. In the fast speed mobile network, location of the blind nodes neighbor will be out of range as the nodes are moving far away from each other in a shorter time. As the previous blind nodes neighbor is out of range, the previous sample will be inadequate to estimate the current blind node location, thus, the new samples are required for the estimation of the current location. Valid samples are harder to obtain when the mobility speed of the sensor node is high, resulting in a repetitive sampling process until enough sample is obtained. The communication cost is low when the mobility speed is high, this causes a less number of nodes being selected for the localization process. Less communication means less energy consumed. Compared to MSL* and LCC, IACS requires the least costs requirement.

DOI value is the variability of radio range’s signal strength and direction of the sensor node. The slightest irregularity of radio range and direction can contribute to the increments of the size of overlap area of radio transmission and reduce the number of neighbors of each node, thus reducing the localization accuracy and communication possibility. From the results (Figure 9c), computation cost is at maximum when DOI value is at 0.3, then it drops at 0.4 and above due to difficulty in obtaining valid samples. As the DOI increases, each node faces difficulties to detect neighbors, thus causing the disability to produce enough valid samples in the localization process. In our simulation, IACS has shown to be more suitable than LCC and MSL* in a high DOI value. IACS also requires the least cost of computation, communication and energy consumption, when compared toLCC and MSL*.

Scalability is one of the crucial issues in designing a WSN network. We consider the scalability as the ability to maintain the cost of the localization process while increasing the size of the network. Therefore, IACS is simulated on different node densities and the results are shown in Section 5.5.5. The accuracy of localization improves as the nodes density increases. This is possible since the localization process of each node depends on the information from its neighbors. As the network expands, all the cost will eventually increase. However, our results show that IACS requires the least cost of computation, communication and energy consumption while maintaining the localization error compared with MSL* and LCC even at a high density of nodes.

Based on the results of the simulation, there are small trade-offs between the cost of communication and computation with the accuracy of the localization methods tested. IACS gain approximately 1.4% more estimation error than LCC with 15% less communication and 13% computation tested using the default parameter stated in the experimental setup section (Section 3) and the results in Section 5.5.1. The methods are also tested in different scenarios. As LCC and IACS work well only when the nodes mobility speed are low and different maximum samples of number do not result in a significant improvement of the accuracy. IACS is proven to work better than MSL* and LCC in a higher DOI value environment. When DOI = 0.5, IACS accuracy is 3.13% better than LCC. In term of node density, IACS obtained a low estimation error in a high node density environment like MSL* and LCC with lower costs. From the discussion, it shows that IACS works very well in high node density, high DOI value, and low maximum velocity node’s environments.

## 7. Conclusions

In this paper, we focused on the conservation of sensor nodes overall localization cost and redundancy management in data sharing and transmission. Our study shows that MSL* uses both seed and normal node to estimate blind node location that is outside the range of seed nodes but within the range of normal nodes. Though the MSL* has the advantage of better localization accuracy, the method will increase the overall communication cost in the network. The MSL* drawback is then mitigated by LCC which reduces the communication cost. However, the filtering process in LCC is exposed to redundancy, where the blind node may have neighbors that share the same information even after the filtering process. Furthermore, LCC neglect the data shared by second hop neighbors, even though the localization process includes the samples from both first and second hop neighbors. Therefore, a method to reduce data redundancy that would affect computation, communication and energy cost of localization process is proposed.

The proposed method, which is named as IACS, is based on the intersection and complement sets that select the most significant neighboring nodes for the localization process. IACS consists of two main steps namely, duplication cleaning and average filtering. In the duplication cleaning step, the neighbor nodes that contain unique intersection and complement sets are selected for the localization process. If the intersection and complement set of normal nodes are identical, the node with the shortest distance to the blind node will be selected. Then, the average filtering step will further filter the nodes selected in the duplication cleaning step based on their total intersection and complement elements. This process excludes the nodes with the total intersection or complement elements that are lower than the average of intersection or complement elements in the neighborhood, respectively. Through these steps, the proposed IACS allows the selection of the most significant nodes to reduce the costs required for localization of the blind node. From the simulation, IACS reduced 25% of computation cost, 25% of communication cost and 6% of energy consumption compared to MSL*, while 15% of computation cost, 13% of communication cost and 3% of energy consumption compared to LCC. Furthermore, IACS also required the least cost of computation, communication and energy in various scenarios tested compared to MSL* and LCC. Based on the research, there are also research gaps found on the MCL-based algorithm that can be done for future works. Most of WSN localization methods are implemented using simulation due to the high cost for the full-scale hardware environment. Furthermore, the resampling process used in the recent PF algorithm does not prevent the samples deterioration [30]. On the other hand, the presence of obstacles and walls in the area can cause the sensor node to be in a Non-line-of-sight (NLOS) state, where the signal transmission between nodes can derange [31]. Both samples deterioration and deranged signal transmission may lead to localization inaccuracy and failures. On that note, future works to efficiently avoid PF samples deterioration, to prevent deranged signal transmission among nodes caused by obstacles or walls, and full-scale hardware implementation could contribute significantly to the field.

## Figures and Tables

**Table 1:** Experimental Setup Parameters.

Parameter	Value
Time Slot	200
Iteration	50
*maxv*	0.2*r*
*r*	50 unit
Environment	Square bordered area of 500 unit × 500 unit
Maximum sample number	50 samples
Mobility model	Modified Random Waypoint with 0 pause time
Base Density	32
Sd	1.0
Nd	10.0

**Table 2:** LCC Intersection set example.

Id	Neighbor Set	Intersection Set
0	{1, 2, 3, 5, 7, 8, 9}	{1, 7, 8, 9}
1	{0, 3, 5, 6, 7, 8, 9}	{0, 6, 7, 8, 9}
6	{1, 3, 4, 5, 8, 9}	{1, 8, 9}
7	{0, 1, 3, 5, 8, 9}	{0, 1, 8, 9}
8	{0, 1, 2, 3, 4, 5, 6, 7}	{0, 1, 6, 7}
9	{0, 1, 2, 4, 5, 6, 7}	{0, 1, 6, 7}

**Table 3:** Sample of Duplicate Intersection Sets extracted from Figure 2. *t* and *n* represent the Time Slot and the Node Id, respectively. The correspondence values between the Time Slot and the Node ID represent the number of duplication detected for the node in each time slot.

	Time Slot (t)	*t*_1_	*t*_2_	*t*_3_	*t­*_4_	*t*_5_	*t*_6_-*t*_195_	*t*_196_	*t*_197_	*t*_198_	*t*_199_	*t*_200_
Node Id (n)												
*n*_1_		1	4	2	5	3	…..	7	10	4	2	5
*n*_2_		2	4	6	3	3	…..	5	7	10	5	8
*n*_3_–*n*_318_		: :	: :	: :	: :	: :	: :	: :	: :	: :	: :	: :
*n*_319_		2	3	2	5	3	…..	2	5	1	8	10
*n*_320_		3	3	1	2	2	…..	6	4	4	5	7

**Table 4:** Basic statistics of duplicate intersection sets for simulation in Figure 2.

Statistical Approach	Value
Minimum	0
Maximum	20
Average	4.76 ≈ 5
Mode	3
Standard deviation	2.81

**Table 5:** Duplication Cleaning Table.

Id	Intersection Set	Intersection Duplicate	Complement Set	Complement Duplicate	Distance to Blind (*m*)	Remove?
0	{1, 7, 8, 9}	No	{2, 3, 5}	No	21	No
1	{0, 6, 7, 8, 9}	No	{3, 5}	Yes (with node 7)	9	No
6	{1, 8, 9}	No	{3, 4, 5}	No	20	No
7	{0, 1, 8, 9}	No	{3, 5}	Yes (with node 1)	10	Yes
8	{0, 1, 6, 7}	Yes (with node 9)	{2, 3, 4, 5}	No	12	No
9	{0, 1, 6, 7}	Yes (with node 8)	{2, 4, 5}	No	15	Yes

**Table 6:** Average Filtering Table.

Id	Intersection Set	Intersection Elements	Complement Set	Complement Elements	Remove?
0	{1, 7, 8, 9}	4	{2, 3, 5}	3	No
1	{0, 6, 7, 8, 9}	5	{3, 5}	2	Yes: Low complement element than average
6	{1, 8, 9}	3	{3, 4, 5}	3	Yes: Low intersection element than average
8	{0, 1, 6, 7}	4	{2, 3, 4, 5}	4	No
Total (Average)		16 (4)		12 (3)	

## List of Acronyms

IACS	Intersection and Complement Set
WSN	Wireless Sensor Network
KF	Kalman Filter
PF	Particle Filter
LCC	Low Communication Cost
MSL*	Mobile and Static sensor network Localization*
ToA	Time of Arrival
TDoA	Time Difference of Arrival
RToF	Return Time of Flight
AoA	Angle of Arrival
RSS	received signal strength
CSI	Channel State Information
MCL	Monte Carlo Localization
GPS	Global Positioning System
MSL	Mobile and Static sensor network Localization
MCB	Monte-Carlo Box
VMSL	V-Mobile and Static sensor network Localization
VMSL*	V-Mobile and Static sensor network Localization*
WMCL	Weighted Monte Carlo Localization
IDE	Integrated Development Environment
2E	2-dimensional Euclidean space
Nd	Node density
Sd	Seed density
Id	Identity number
NLOS	Non-line-of-sight
